# Remote Musculoskeletal Consultations: A Survey of General Practitioner Registrars’ Level of Confidence, Acceptability, and Management

**DOI:** 10.7759/cureus.15084

**Published:** 2021-05-18

**Authors:** Manroy Sahni, Jamaal Choudhry, Ankush Mittal, Gurjit Bhogal

**Affiliations:** 1 Family Medicine, Royal Wolverhampton NHS Trust, Wolverhampton, GBR; 2 Orthopaedics, Royal Wolverhampton NHS Trust, Wolverhampton, GBR; 3 Department of Public Health, City of Wolverhampton Council, Wolverhampton, GBR; 4 Physical Medicine and Rehabilitation, Centre for Musculoskeletal Medicine, Royal Orthopaedic Hospital, Birmingham, GBR

**Keywords:** general practice, remote consultation, remote msk examination, telemedicine, general practice registrar

## Abstract

Background and objective

The coronavirus disease 2019 (COVID-19) pandemic has accelerated the shift towards remote consultations in the medical field, including musculoskeletal (MSK) appointments. General practitioner (GP) registrars are now routinely conducting many MSK consultations remotely; however, very little is known of their level of confidence and satisfaction regarding this new and evolving scenario, or how this may impact patient management of patients. In this study, we aimed to understand GP registrars' level of confidence and satisfaction with respect to remote MSK consultations, and the perceived impact on patient management.

Study design

This study involved a cross-sectional online survey of GP registrars in the West Midlands, which was conducted in January 2021.

Methods

The survey asked for ranked responses to questions comparing face-to-face consulting methods with remote consulting, focusing on confidence, satisfaction, onward investigations, and referral activity. Statistical analysis was performed using the R software version 4.0.3.

Results

The overall survey response was 21.2% (n=312/1,471). Of the respondents, 85.9% of GP registrars had not received any training to prepare them for remote MSK consultations. GP registrars generally felt that they were more confident when treating patients face-to-face compared to remote consultations (p<0.001). This was true for general MSK complaints as well as specific assessments of the hand, shoulder, spine, hip, knee, and ankle; 36.2% of GP registrars were not satisfied and 51.0% thought that their patients were not satisfied with the current quality of remote MSK consultations. Of note, 77.6% of GP registrars said that they were more likely to request additional investigations, and 75.6% stated that they were more likely to refer patients to a specialist after a remote MSK consultation.

Conclusion

This study highlights the need for further training to better equip primary care doctors for remote MSK consultations. With tailored training, GP registrars could offer more streamlined remote patient care for MSK complaints.

## Introduction

Musculoskeletal (MSK) complaints account for more than 100 million general practitioner (GP) appointments each year in the UK; they constitute almost 30% of the caseload and cost the National Health Service (NHS) £5 billion per year [1]. An ageing population means that these numbers are expected to increase further, with MSK conditions already recognised as the single greatest contributor to the country’s growing burden of disability [2]. Hence, it is of paramount importance that GPs, who often represent the gateway to the NHS, are equipped to effectively diagnose and manage MSK presentations.

However, the literature indicates that current medical school curriculums do not impart satisfactory levels of knowledge and confidence with respect to treating MSK conditions [3,4]. Unsurprisingly, this same theme is echoed further along the medical training pathway for both primary and secondary care doctors [5,6]. Abou-Raya et al. have concluded that "the time devoted to rheumatology education and training is disproportionately low compared to the frequency of musculoskeletal complaints encountered in general practice" [6].

To address these issues and better streamline MSK care, Health Education England and NHS England Medical Directorate have commissioned the development of an MSK core capabilities framework [7]. This framework aims to transform MSK services in England by placing skilled MSK practitioners earlier in the patient pathway. In addition to these positive steps, the growing availability and popularity of virtual MSK consultations offer enhanced accessibility and the potential to overcome traditional barriers to presentation [8,9].

Due to the unprecedented nature of the coronavirus disease 2019 (COVID-19) pandemic and the consequent necessity of social distancing measures, many GP consultations are now taking place remotely, either over the telephone or via video consultation platforms [10]. Moreover, the Chair of the Royal College of General Practitioners (RCGP) has stated that as many as half of GP consultations may be carried out remotely even after the COVID-19 pandemic has passed [11]. For GP registrars, the COVID-19 pandemic has had a significant impact on training, which includes alterations to the format of Royal College examinations, and a recent report has estimated that as many as 90% of GP consultations are currently being undertaken remotely [12].

Ultimately, the pandemic has forced the NHS to rethink its care models and cater to new ways of living, and it appears that remote consultations are here to stay. However, many questions remain unanswered regarding this new way of working, not least for primary care-based MSK care, which has historically involved frequent physical examinations. GP registrars, who represent the future of community medicine, have been thrust into a dynamic sphere of remote consultation, but the pertinent question is as follows: how confident are they when it comes to conducting MSK assessments in this manner and how will it affect their practice? We aimed to address these challenges in this study by analysing GP registrars' level of confidence and satisfaction with respect to remote MSK consultations, and the perceived impact on patient management.

## Materials and methods

Study design and aims

Through an online cross-sectional survey, this study aimed to gauge the confidence and satisfaction levels among GP registrars regarding remote MSK consultations. The survey also aimed to examine whether remote consulting impacts the onward management of MSK patients, specifically with regard to requesting investigations and onward referral to secondary care. Likert scales were used for ease of completion, consistency, and objective comparison against face-to-face consultation. The online survey design facilitated effective distribution to GP registrars across the West Midlands during the COVID-19 pandemic and allowed for a large sample size. Data collection was conducted in a fully anonymous manner, and GP registrars completed the survey at their convenience.

Setting and participants

GP registrars of all grades (ST1, ST2, and ST3) were included in this study. GP registrars are qualified doctors enrolled in the specialty training programmes overseen by the RCGP. The survey was distributed across the West Midlands Deanery, to all five GP schools in the region (Coventry and Warwickshire, Black Country, Birmingham and Solihull, Staffordshire and Shropshire, and Hereford and Worcestershire). GP registrars were included if they were currently in training and also if they were taking time out of the programme for any reason, including health issues, maternity/paternity leave, or academic activity. Fully qualified GPs were excluded.

Survey development

Survey questions were based on a questionnaire used in a recent study assessing the confidence of medical students with regard to their MSK curriculum and teaching [13]. The questions were adapted and modified to be in line with the aims of this study before being scrutinised by a group of eight experts. The eight experts had completed the GP training pathway, prior to specialising in Sports and Exercise Medicine. They were therefore familiar with the job role of the GP registrars and had considerable experience in the MSK field.

The survey was created using the online tool SurveyMonkey® (http://www.surveymonkey.com; SVMK Inc., San Mateo, CA).

The survey materials used in this study are available from the authors on request.

Survey piloting

Following expert review, the survey was piloted among a sample of 10 West Midlands GP registrars. These survey responses were not included in the final analysis. Based on the feedback from the pilot, minor adjustments were made to the wording of the questions to provide more clarity.

Survey distribution

Prior to the distribution, approval for the survey was obtained from the Head of School for GP Education in the West Midlands. The survey link was then emailed to GP registrars across the West Midlands from the Faculty Support Team at the Deanery. A short explanation was provided in the email, detailing the composition of the research team, and explaining the purpose of the survey. It was made clear that participation was completely voluntary and that responses would be anonymous. All participants had the option to ask the research team any questions prior to completing the survey. The same message and survey link were circulated to GP registrars through peer support groups and trainee representatives. A reminder email was sent by the Faculty Support Team one week later. The survey was kept open for two weeks in January 2021.

Data management and statistical analysis

Anonymity was ensured regarding survey responses. Questions on the level of confidence were scored on a scale of 1-5 (1 signifying "not at all confident", 3 signifying "neutral", and 5 signifying "very confident"). Questions on the level of satisfaction were also scored on a scale of 1-5 (1 signifying "not at all satisfied", 3 signifying "neutral", and 5 signifying "very satisfied"). Simple descriptive statistics and bar charts were used to present overall responses for key domains. The Wilcoxon signed-rank test was used to assess if there was a statistically significant difference between responses in terms of remote versus face-to-face consultations. Statistical analysis was performed using the R software version 4.0.3.

Ethical considerations 

The Health Research Authority confirmed that no ethical approval was required for this anonymous staff survey. The project was locally approved by the Royal Orthopaedic Hospital, Birmingham.

## Results

The survey was distributed to a total of 1,471 GP registrars across the West Midlands. We received responses from 312 GP registrars and all those were included in the final analysis (response rate: 21.2%).

Participant characteristics

Of the 312 participants, 57 (18.3%) were in specialty training year ST1, 98 (31.4%) were in ST2, and 157 (50.3%) were in ST3, which represents the final year before qualification. When asked how many remote consultations for MSK complaints they were doing per week, 33 (10.6%) stated they were doing 20 or more, 104 (33.3%) were doing between 10 and 19, 133 (42.6%) were doing 1-10, and 42 (13.5%) were doing none.

Of the 312 participants, 268 (85.9%) stated that they had not received any form of training to prepare them for remote MSK consultations. Of the 44 (14.1%) who had received prior training, the most commonly stated sources of education were informal teaching from supervisors, self-directed learning, and scheduled GP teaching sessions.

Level of confidence

**Figure 1:**
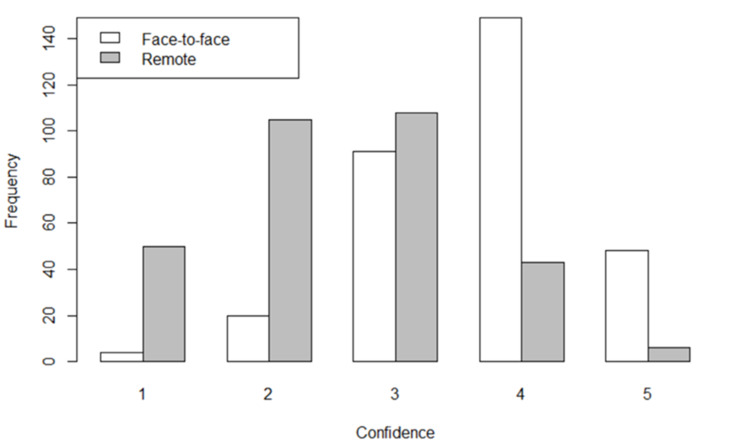
GP registrars' self-reported confidence level when treating patients with MSK complaints face-to-face versus remotely (n=312) 1 signifying "not at all confident", 2 signifying "not confident", 3 signifying "neutral", 4 signifying "confident", and 5 signifying "very confident" GP: general practitioner; MSK: musculoskeletal

With regard to treating patients with MSK complaints face-to-face, 197 (63.1%) registrars reported feeling "confident" or "very confident" (score of 4 or 5). In comparison, only 49 (15.7%) registrars felt "confident" or "very confident" when treating patients with MSK complaints remotely (Figure 1). GP registrars reported significantly more confidence when treating patients face-to-face compared to remote consultations (p<0.001).

For each of the six regions evaluated (ankle, knee, hip, spine, shoulder, and hand), GP registrars were more confident assessing the patient face-to-face compared to remotely (p<0.001) (Table 1).

**Table 1:** GP registrars' self-reported confidence level when assessing joint complaints face-to-face versus remotely (n=312) GP: general practitioner

Variables		Responses, % (n)					P-value (face-to-face vs. remote)
Joint being assessed	Consultation type	1 (not at all confident)	2	3 (neutral)	4	5 (very confident)	
Ankle	Face-to-face	1.28 (4)	7.37 (23)	28.53 (89)	50.64 (158)	12.18 (38)	<0.001
	Remote	25.00 (78)	36.22 (113)	30.13 (94)	7.69 (24)	0.96 (3)	
Knee	Face-to-face	1.92 (6)	4.49 (14)	19.56 (61)	52.24 (163)	21.79 (68)	<0.001
	Remote	20.83 (65)	37.50 (117)	28.53 (89)	12.18 (38)	0.96 (3)	
Hip	Face-to-face	1.60 (5)	4.49 (14)	19.55 (61)	51.28 (160)	23.08 (72)	<0.001
	Remote	22.44 (70)	37.50 (117)	30.77 (96)	8.33 (26)	0.96 (3)	
Spine	Face-to-face	1.28 (4)	4.48 (14)	22.44 (70)	48.08 (150)	23.72 (74)	<0.001
	Remote	20.83 (65)	44.24 (138)	26.60 (83)	7.05 (22)	1.28 (4)	
Shoulder	Face-to-face	1.28 (4)	6.73 (21)	23.08 (72)	44.87 (140)	24.04 (75)	<0.001
	Remote	18.27 (57)	41.67 (130)	29.49 (92)	8.97 (28)	1.60 (5)	
Hand	Face-to-face	1.28 (4)	6.73 (21)	22.12 (69)	46.47 (145)	23.40 (73)	<0.001
	Remote	18.59 (58)	41.03 (128)	29.81 (93)	8.97 (28)	1.60 (5)	

Acceptability

**Figure 2:**
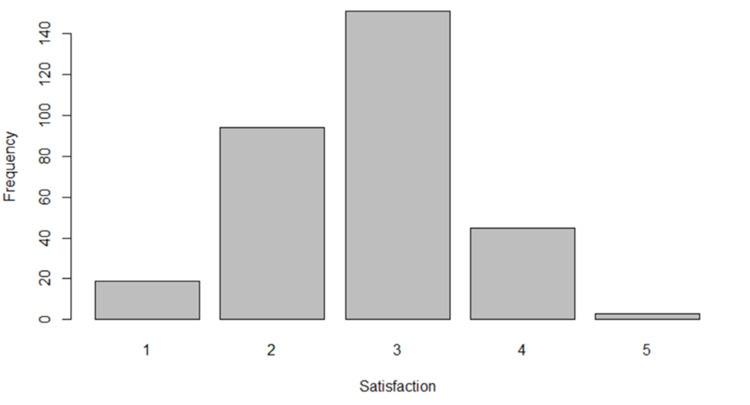
Clinician satisfaction with the current quality of remote MSK consultations (n=312) 1 signifying "not at all satisfied", 2 signifying "not satisfied", 3 signifying "neutral", 4 signifying "satisfied", and 5 signifying "very satisfied" MSK: musculoskeletal

When asked for their opinion on the current quality of remote MSK consultations, 113 (36.2%) GP registrars were "not satisfied" or "not at all satisfied" (score of 1 or 2), whereas only 48 (15.4%) GP registrars were "satisfied" or "very satisfied" (score of 4 or 5) (Figure 2).

**Figure 3:**
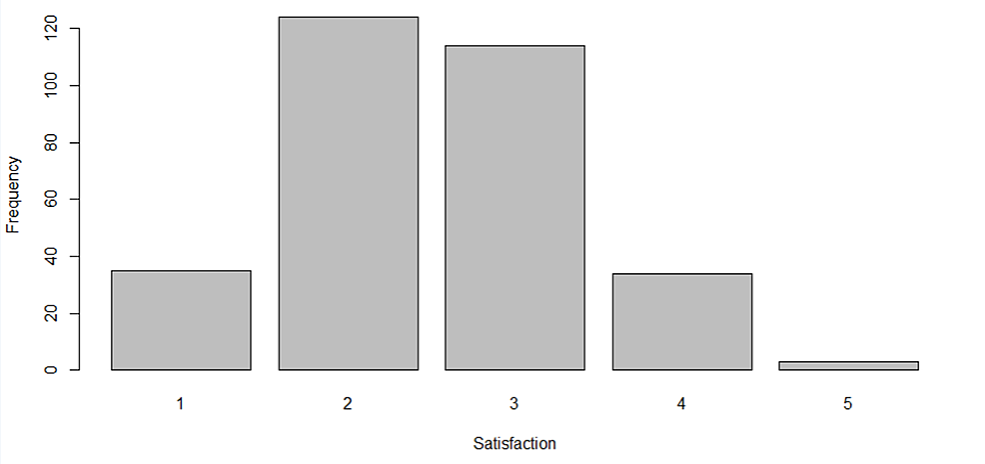
Perceived patient satisfaction with the current quality of remote MSK consultations as reported by GP registrars (n=312) 1 signifying "not at all satisfied", 2 signifying "not satisfied", 3 signifying "neutral", 4 signifying "satisfied", and 5 signifying "very satisfied" GP: general practitioner; MSK: musculoskeletal

When asked what they thought their patients’ opinions were regarding the current quality of remote MSK consultations, 159 (51.0%) GP registrars thought their patients were either "not satisfied" or "not at all satisfied" (score of 1 or 2). In comparison, only 37 (11.9%) GP registrars thought their patients were "satisfied" or "very satisfied" (score of 4 or 5) (Figure 3).

Management

Of note, 242 (77.6%) GP registrars reported they were more likely to request further investigations following a remote MSK consultation (for example, blood tests or imaging) compared to face-to-face appointments. Furthermore, 236 (75.6%) GP registrars reported they were more likely to refer the patient to see a specialist after a remote consultation in contrast with face-to-face patient interactions.

## Discussion

Summary

This study found that the West Midlands GP registrars were significantly more confident when treating patients face-to-face compared to remote consultations (p<0.001). This was true for general MSK complaints as well as more specific assessments of the hand, shoulder, spine, hip, knee, and ankle. Regarding acceptability, 36.2% of GP registrars were not satisfied and 51.0% thought that their patients were not satisfied with the current quality of MSK consultations. GP registrars also reported that remote consulting for MSK complaints would prompt them to request additional investigations at a significantly higher rate (77.6%) and would make them more likely to refer patients on to a specialist (75.6%).

Strengths and limitations

To the authors’ knowledge, this is the first study to evaluate the level of confidence, acceptability, and management choices of GP registrars in the context of remote MSK consultations. A relatively large sample size was achieved for this study within one of the largest GP schools across the country. Given the widescale shift towards remote consulting, accelerated by the COVID-19 pandemic and social distancing requirements, it is vital to understand the views of clinicians and how practice may be altered. This is particularly important with respect to MSK complaints, where historically the importance of the "hands-on" physical examination has been emphasised.

The response rate of 21.2% represents a fair return for an online survey conducted during a pandemic, when the healthcare professionals are dealing with unprecedented levels of workload and are at an increased risk of burnout [14]. Furthermore, it is well-known that primary care doctors tend to be poor responders to web-based surveys [15]. Despite this, we understand that this response rate may increase the chances of selection bias, and it would be critical in future studies to detail responder characteristics to ensure that they fairly represent the wider target population. Involving a panel of experts, with a background in both general practice and MSK medicine, during the survey design enhanced the validity and relevance of the questions asked.

The survey was distributed to all GP registrars within the West Midlands Deanery, which is one of the largest schools of general practice in the UK. To build upon this study, it would be beneficial to distribute the survey to all GP schools across the country to achieve a fairer representation of GP registrars. Furthermore, expanding the survey population to include qualified GPs, physiotherapists, and specialist practitioners would offer a more complete picture of the impact of remote MSK across primary care, which is very much multidisciplinary in its orientation and approach [7].

Although detailed demographic data for survey responders were not collected in this study, the large sample size and the degree of significance that were demonstrated likely represent the views of the target population. When considering the level of satisfaction with remote MSK consultations, this study assessed perceived patient satisfaction without consulting patients directly. Engaging with patient groups directly to understand their experiences is an area of further research interest. Furthermore, although outcome data were not collected, this study provides a template to measure management outcomes such as investigation patterns, referral activity, and other clinical outcomes through a formal cohort study.

Comparison with existing literature

Even before the COVID-19 pandemic, remote consultations in primary care were considered an opportunity to offer a time-saving alternative when a formal physical examination was not required [16]. The idea of remote consultations has generally been well received by clinicians and patients in primary care [16-18]. However, some studies have highlighted concerns from GPs regarding issues such as confidentiality, quality of technology, and the impact on diagnostic accuracy [19,20].

As the COVID-19 pandemic has continued to spread and expand, clinicians have been doing significantly more remote consultations, and reports have emerged indicating a lack of clinician confidence [21]. A recent mixed-methods study evaluated the experiences of GP registrars when undertaking telephone consultations and found that "complex encounters" and the "absence of examination" were reported as key drawbacks in remote assessments [22]. The study concluded that GP registrars required further guidance and training to better prepare them for remote telephone consultations. This conclusion is supported by the findings of this study, with lower confidence reported when assessing MSK complaints remotely and 85.9% of GP registrars stating that they had not received any form of training to prepare them for remote MSK consultations. This study underlines that for MSK complaints, there is likely to be a widespread need for enhanced training and support to improve the experience of remote consultations for both doctors and patients.

Literature is now emerging to offer guidance and strategies to effectively implement remote primary care consultations [23,24]. Furthermore, guidance specific to remote MSK consultations is now being developed, such as virtual joint assessment guides and solutions to enable clinicians to provide satisfactory patient care [25-27]. Such resources may help to address the poor satisfaction levels and tendency to order more investigations and refer more MSK patients to see a specialist as observed in this study.

## Conclusions

We believe this study amplifies the call for the development of training interventions for clinicians undertaking remote consultations. Our findings endorse the case for implementing postgraduate medical training programmes to prepare doctors for remote MSK consultations, and more specifically within the RCGP curriculum. With tailored training, GP registrars could offer more streamlined patient care for MSK complaints, which would potentially lead to fewer unnecessary investigations as well as a reduced number of referrals to secondary care specialists.

Further research is needed to better understand the viewpoints of GP registrars and ascertain why they feel less confident when treating MSK complaints remotely. This, coupled with similar research from a patient perspective, would enable the development of holistic education programmes to better prepare clinicians for remote MSK consultations. Employing a qualitative methodology would further allow for in-depth exploration of the reasons for lower confidence and dissatisfaction with remote consultation as reported in this study. Collectively, such evidence could be used to develop and enhance relevant learning resources, as well as guide the development of digital interfaces to assist doctors with remote MSK consultations in real time. We believe further research into the themes uncovered in this study will contribute significantly to meeting the MSK health needs of our population as we prepare to come to terms with a more digital NHS.
